# An improved protein lipid overlay assay for studying lipid–protein interactions

**DOI:** 10.1186/s13007-020-00578-5

**Published:** 2020-03-06

**Authors:** Xiuli Han, Yongqing Yang, Fengyun Zhao, Tianren Zhang, Xiang Yu

**Affiliations:** 1 College of Life Sciences, Shandong University of Technology, Zibo, 255049 China; 2 State Key Laboratory of Plant Physiology and Biochemistry, College of Biological Sciences, China Agricultural University, Beijing, 100193 China

**Keywords:** Protein lipid overlay, Lipid–protein interactions, PLO

## Abstract

**Background:**

Lipids perform multiple functions in the cell, and lipid–protein interactions play a key role in metabolism. Although various techniques have been developed to study lipid–protein interactions, the interacting protein partners that bind to most lipids remain unknown. The protein lipid overlay (PLO) assay has revealed numerous lipid–protein interactions, but its application suffers from unresolved technical issues.

**Results:**

Herein, we found that blocking proteins may interfere with interactions between lipids and their binding proteins if a separate blocking step is carried out before the incubation step in the PLO assay. To overcome this, we modified the PLO assay by combining an incubation step alongside the blocking step. Verification experiments included phosphatidylinositol-3-phosphate (PI3P) and its commercially available interacting protein G302, C18:1, C18:2, C18:3 and the Arabidopsis plasma membrane H^+^-ATPase (PM H^+^-ATPase) AHA2 C-terminus, phosphatidylglycerol (PG) and AtROP6, and phosphatidylserine (PS) and the AHA2 C-terminus. The lipid–protein binding signal in the classical PLO (CPLO) assay was weak and not reproducible, but the modified PLO (MPLO) assay displayed significantly improved sensitivity and reproducibility.

**Conclusions:**

This work identified a limitation of the CPLO assay, and both sensitivity and reproducibility were improved in the modified assay, which could prove to be more effective for investigating lipid–protein interactions.

## Background

Lipids were previously thought to function as barriers that generate compartments in cells, but recent studies reveal other functions in addition to acting as physical membrane bilayer and structural components [21, 36]. Lipids also function in signal transduction, trafficking, morphological changes, and cell division via specific lipid–protein interactions [3, 11, 16, 21, 35]. The study of lipid–protein interactions can uncover the functions of lipids in the cell, and systematic analysis has reportedly come of age [28, 33]. Various techniques have been developed to study lipid–protein interactions, such as nuclear magnetic resonance spectroscopy (NMR) [2, 30], isothermal titration calorimetry (ITC) [24, 32], surface plasmon resonance (SPR) [6, 26], liposome sedimentation assay [12, 31], protein lipid overlay (PLO) assay [18, 29, 30], microscale thermophoresis (MST) [7, 34], and native mass spectrometry [36]. To explore more unknown functions of diverse lipid species, new and/or improved techniques are essential to probe lipid–protein interactions.

The PLO assay is a technique widely used to study lipid–protein interactions [1, 8, 23, 30]. Although commercially available lipid-attached membranes can be purchased by Echelon (https://www.echelon-inc.com/) and a detailed protocol is supplied, the PLO assay has limitations that must be overcome. In a classical PLO (CPLO) assay, membranes are spotted with lipids, followed by a serial procedures including blocking with blocking proteins, incubation with epitope-tagged target proteins, incubation with primary antibodies, incubation with secondary antibodies, and detection with ECL western reagent [8]. A western blotting assay is performed similarly as a CPLO assay, except that membranes are coated with proteins and followed by incubation and detection procedures [14]. The very similar protocols indicate that CPLO assays may be treated similarly to western blot assays, although the types of molecules detected differ, not least in terms of molecular mass (low for lipids and high for proteins). Previous studies using CPLO assays have mainly focused on partial lipid species such as PIP or PIP2 [16, 20, 30], whereas larger species such as SIP, PG, and PS are less well-studied. Whether CPLO assays can be improved to facilitate studying a wider variety of lipid–protein interactions requires investigation.

In our present study, we found that the blocking procedure using blocking proteins such as bovine serum albumin (BSA) or skimmed milk proteins can cause steric hindrance for the binding of membrane lipids to their binding protein partners, if a separate blocking step is carried out before the incubation step. To avoid hindrance by blocking proteins, we modified the PLO assay by combining an incubation step alongside the blocking step based on the principle that the specific bindings between lipids and their binding proteins are more stronger than the non-specific bindings between membrane and blocking proteins, and between lipids and blocking proteins.

The resulting modified PLO (MPLO) assay is described in the detail in this study. Because lipids are small amphiphilic molecules with complex solubility behavior that can be unstable if stored improperly (8), we also addressed how best to dissolve and store lipids for both PLO assays and cell- or protein-compatible activity assays. Verification experiments included phosphatidylinositol-3-phosphate (PI3P) and its commercially available interacting protein G302, C18:1, C18:2, C18:3 and the Arabidopsis plasma membrane H^+^-ATPase (PM H^+^-ATPase) AHA2 C-terminus, phosphatidylglycerol (PG) and AtROP6, and phosphatidylserine (PS) and the AHA2 C-terminus.

Lipids perform both structural and cell signaling functions, but their signaling functions are less well-studied. The MPLO assay developed herein could be applied for a wider variety of lipids, including those with smaller head groups, to investigate their functions in plant, animal, and microbial cells.

## Materials and methods

### Chemicals reagents

The lipids used were the same as previously reported [12], briefly, PC (phosphatidylcholine), PE (phosphatidylethanolamine), PG, PA (phosphatidic acid), DG (diglyceride) and PI(3)P were ordered from Avanti Polar Lipids, Inc. with catalog numbers of 850375, 850725, 841148, 840875, 800811 and 850150 respectively; LPA (lysophosphatidic acid) and MG (monoglyceride) were ordered from Sigma-Aldrich with catalog numbers of L7260 and M7765 respectively; MGDG (monogalactosyldiacylglycerol), DGDG (digalactosyldiacylglycerol), SQDG (sulfoquinovosyl diacylglycerol), PS and PI (phosphatidylinositol) were ordered from Lipid Products, UK; CCCP (Carbonyl cyanide 3-chlorophenylhydrazone, catalog number C2759) was ordered from Sigma-Aldrich. G302 was ordered from echelon; Bradford (catalog number #500-0205) was ordered from BIO-RAD. Polyvinylidene difluoride (PVDF) membrane (0.45 µm) was ordered form Millipore USA.

### Plant material and growth conditions

*Arabidopsis thaliana* Columbia (Col-0) seeds were sterilized in a solution containing 20% (v/v) sodium hypochlorite and 0.1% (v/v) Triton X-100 for 10 min, washed with sterilized distilled water for five times, sown on Murashige and Skoog salts (MS, Sigma-Aldrich) medium containing 20 g/L sucrose and 3 g/L phytagel (Sigma-Aldrich), kept at 4 °C in the dark for 3 days, and grown in a controlled growth chambers under 16-h light (22 °C)/8-h dark (20 °C) cycle for 8 days (light intensity of 50 μmol m^–2^ s^–1^). Then the seedlings were transferred to a conventionally fertilized soil in a growth room with a cycle of 16 h light (light intensity of 50 μmol m^–2^ s^–1^) at 22 °C and 8 h dark at 20 °C. Plants were watered three times a week. After 4 weeks, the seedlings were collected for further plasma membrane vesicles isolation and PM H^+^-ATPase activity detection.

### Isolation of plasma membrane vesicles and PM H^+^-ATPase activity assays

Plasma membrane-enriched vesicles were isolated from Arabidopsis seedlings by aqueous two-phase (Dextron-PEG3350) partitioning method [27]. The seedlings collected were homogenized in isolation buffer (2 mL buffer per g plant tissue containing 10% (w/v) glycerol, 5 mM EDTA, 0.2% (w/v) casein, 0.33 M sucrose, 0.2% (w/v) BSA, 5 mM DTT, 5 mM ascorbate, 0.6% (w/v) polyvinylpyrrolidone, 1× protease inhibitor, 1 mM PMSF, and 50 mM HEPES–KOH, pH 7.5). The homogenate was filtered through Miracloth and applied for centrifugation at 12,000*g* for 10 min at 4 °C. The supernatant was transferred out and further applied for centrifugation at 100,000*g* at 4 °C for 1 h. The microsomal pellet was reserved, re-suspended in buffer I (5 mM K_2_HPO_4_-KH_2_PO_4_, 3 mM KCl, 1 mM DTT, 0.33 M sucrose, 1 mM PMSF, 0.1 mM EDTA, 1× protease inhibitor, pH 7.8), and added into a two-phase mixture (Two-phase mixture: 5 mM K_2_HPO_4_-KH_2_PO_4_, 3 mM KCl, 0.33 M sucrose, 6.2% (w/w) Dextran T-500, and 6.2% (w/w) polyethylene glycol 3350, buffer, pH 7.8) with a ratio of 1:3 (w/w). The upper phase collected from the two-phase mixture was then diluted with the re-suspension buffer II (Re-suspension buffer II: 20 mM HEPES–KOH, 2 mM DTT, 0.33 M sucrose, 0.1% (w/v) BSA, 0.1 mM EDTA, 10% (w/v) glycerol, 1× protease inhibitor, pH 7.5) and applied centrifugation at 100,000*g* at 4 °C for 1 h. The microsomal pellet was reserved, re-suspended in resuspension buffer II, supplemented with 1 mM EDTA to get plasma membrane-enriched vesicles. 0.05% (w/v) Brij58 was then added to produce the inside-out vesicles as described previously [15].

PM H^+^-ATPase activity was detected as described previously [27]. Briefly, the PM H^+^-ATPase activity assay buffer (PM H^+^-ATPase activity assay buffer: 3 mM MgSO_4_, 100 mM KCl, 25 mM 1,3-bis [Tris (hydroxylmethyl) methylamino] propane-HEPES, 250 mM mannitol, 3 mM ATP, 5 mM quinacrine (a pH-sensitive fluorescent probe), pH 6.5) was equilibrated and detected in a fluorescence spectrophotometer (Hitachi F-7000) with the excitation wavelength of 430 nm and the emission wavelength of 500 nm. After the addition of 3 mM of ATP, the PM H^+^-ATPase activity was evaluated based on the decrease of the fluorescent signal. The carbonyl cyanide 3-chlorophenylhydrazone (CCCP, a final concentration of 10 mM) was then used to quench the reaction and the decrease of the fluorescent signal was rescued.

### Structure simulation and calculation

Lipid structure simulation and calculations were performed as previously reported [4, 19]. Briefly, the molecular structures of lipids were drawn using ChemBioDraw Ultra 12.0, transferred to the ChemBio3D Ultra 12.0 module, and pre-optimized using the MM2 force field calculation until the minimum rms error was < 0.100 kcal/mol/Å to generate the lowest energy conformation. Distances between atoms in the energy-minimized lipids were then measured by the tools in the ChemBio3D module. Specifically, one atom was selected in ChemBio3D, the shift key was held, another atom was selected, the distance was measured, distance measurements were displayed.

### Cloning, expression, and purification of AHA2 peptide constructs and AtROP6 protein construct

The cloning, expression, and purification of AHA2 central loop, AHA2 C-terminus, AtROP6, CKL2, and MPK6 were performed similarly as previously reported [12, 13]. Briefly, the coding sequences of *AHA2* central loop, *AHA2* C-terminus, *CKL2*, and *MPK6* from Arabidopsis were amplified by RT-PCR with the primer of L-Bf/L**-**Er for *AHA2* central loop, C-Bf/C-Er for *AHA2* C-terminus, CKL2-Bf/CKL2-Er for *CKL2*, and MPK6-Bf/MPK6-Hr for *MPK6*, respectively. The PCR products were cloned into *pET-28a-SMT3* vector between the *BamH*I and *EcoR*I sites to generate the recombinant plasmid *pET-28a-SMT3-AHA2-central loop*, *pET-28a-SMT3-AHA2*-*C-terminus*, or *pET-28a-SMT3-CKL2*, respectively, or cloned into *pET-28a* vector between the *BamH*I and *Hind*III sites to generate the recombinant plasmid *pET-28a –MPK6*. The coding sequence of AtROP6 was amplified by RT-PCR with the primer of R6Bf/R6Hr, and the PCR products were then cloned into the *pET-30a* vector between the *BamH*I and *Hind*III sites to generate the recombinant plasmid *pET-30a-AtROP6*. The plasmids with the right sequence after sequencing were transformed into *E. coli* strain BL21, and the recombinant proteins were expressed after induction by IPTG and purified using Ni-beads according to manufacturer’s instructions.

### PLO assay

The steps in the MPLO assay and the CPLO assay were performed similarly, except the step 5.

#### Step 1 Lipid packaging

Commercially available lipids were bought from Avanti Polar Lipids (UK), Lipid Products (UK), or Sigma-Aldrich in dry powder or oil form, or solution form dissolved in organic solvent. Lipids with different head groups and different fatty acid chains usually have different solubility; for example, C18:1-PA can be dissolved in both chloroform and methanol (MeOH), but C18:0-PA can only be dissolved in chloroform and not MeOH, and PE from bovine brain cannot be dissolved in either chloroform or MeOH. Solvent 1 (65:35:8 chloroform:MeOH:H_2_O) and solvent 2 (65:25:4 chloroform:MeOH:H_2_O) are good solvents that can dissolve many kinds of lipids.

Some lipids may degrade or oxidize during repeated used, hence it is necessary to prepare small aliquots to ensure quality. If lipids are ordered in dry powder or oil form, they can be dissolved in solvent 1 or solvent 2, then separated into numerous small aliquots in Eppendorf tubes. Notably, chloroform in solvent 1 and solvent 2 may dissolve a small amount of plastic from the Eppendorf tube, hence negative control binding and activity experiments are essential. If lipids are received in solution form, separation can be performed directly. Because organic compounds are usually stable under dry, low temperature, and oxygen-free conditions, it is essential to remove solvent by nitrogen gas or vacuum pump, and they should be stored at − 80 °C or − 20 °C. If a laboratory has argon, filling Eppendorf tube with this gas before lipid storage is advisable.

#### Step 2 Lipid dissolution

For the PLO assay, stored lipid was dissolved in solvent 1 or solvent 2 at an initial concentration of 10 mg/mL. Dimethylsulfoxide (DMSO) is not suggested for PLO assays because it is not easy to remove from the membrane due to its high boiling point (189 °C). Notably, not only solvent 1 and solvent 2, other solvents such as MeOH, chloroform, dichloromethane, or the mixtures of these solvents are also applicable for dissolving lipids, which depends on the type of lipids, and the type of their fatty acid chains, such as C18:1-PA could also be dissolved in chloroform, MeOH, and solvent 1. Notably, solvent 1 and solvent 2 are generally not suitable for biological experiments because chloroform is hazardous to cells and proteins, whereas solvents such as MeOH or DMSO are more appropriate. Lipids can then be serially diluted using the same solvent. For PLO assays, whether or not binding occurs between a lipid and a protein should first be determined by making a series of dilutions (e.g., 10 mg/mL, 1 mg/mL, 0.1 mg/mL, and 0.01 mg/mL), and if binding occurs within the concentration range, additional dilutions should be prepared and tested (e.g., 1 mg/mL, 0.5 mg/mL, 0.2 mg/mL, and 0.1 mg/mL).

#### Step 3 Lipid spotting

Different kinds of lipids or lipids at different dilutions are spotted onto a polyvinylidene fluoride (PVDF) membrane, the position of spotting is marked with a pencil, and spots should be separated by ~ 0.7 cm. Spots should be ~ 0.5–1 µL in volume, and a 2 µL pipette or glass capillary can be used to perform spotting. A glass capillary performs better and suffers less from solvent evaporation, and can perform spotting onto many pieces of PVDF membrane at the same time. However, when using a glass capillary, the exact volume of lipid spotted is not known, but our experiments indicate that the volume spotted had no significant influence on the signal detected, whereas the concentration of the lipid solution played a major role in this process. Thus, the difference in signal detected between a spotting volume of 0.5, 1, or 2 µL is not significant; only the radius of the spot changes.

#### Step 4 Lipid drying

Membranes spotted with lipids are dried at room temperature for 30 to 60 min.

#### Step 5 Blocking and incubation

For MPLO assays, fresh PBST blocking buffer (0.1% Tween 20, v/v) containing BSA (3%, m/v) or PBST (0.1% Tween 20, v/v) containing skimmed milk (5%, m/v) is prepared, epitope-tagged (GST, MBP, His, etc.) proteins of interest at 1–5 µg/mL are added, and the membrane is blocked and incubated at 4 °C overnight or at room temperature with gentle agitation for 1.5 h. Notably, fresh blocking buffer is important since stale buffer may lead to experimental failure, and no signal detected. The volume of blocking buffer containing epitope-tagged proteins will depend on the method used to block and incubate the membrane; if using a sealed plastic bag, 500 µL to 1 mL is usually sufficient, but some containers require a larger volume.

For CPLO assays, PVDF membranes spotted with lipids are first blocked with blocking buffer for 1 h, then incubated with blocking buffer containing epitope-tagged (GST, MBP, His, etc.) proteins of interest for another hour.

#### Step 6 Washing

Membranes are washed four times with PBST for 10 min each time.

#### Step 7 Incubation with primary antibody

Membranes are incubated with primary antibody that recognizes the epitope of the lipid-binding protein in blocking buffer at room temperature for 1 h.

#### Step 8 Washing

Membranes are washed another four times with PBST for 10 min each time.

#### Step 9 Incubation with secondary antibody

Membranes are incubated with a horseradish peroxidase (HRP)-conjugated secondary antibody that recognizes the primary antibody in blocking buffer at room temperature for 1 h.

#### Step 10 Washing

Membranes are washed another four times with PBST for 10 min each time.

#### Step 11 Detection

Lipid-binding proteins bound to the membrane are detected by enhanced chemiluminescence (ECL) according to the manufacturer’s instructions. Please note that it is easy to overexpose the membrane during this step, and it is recommended to dilute the ECL reagent before first use.

## Results

### Overview of the MPLO assay procedure

**Fig. 1:**
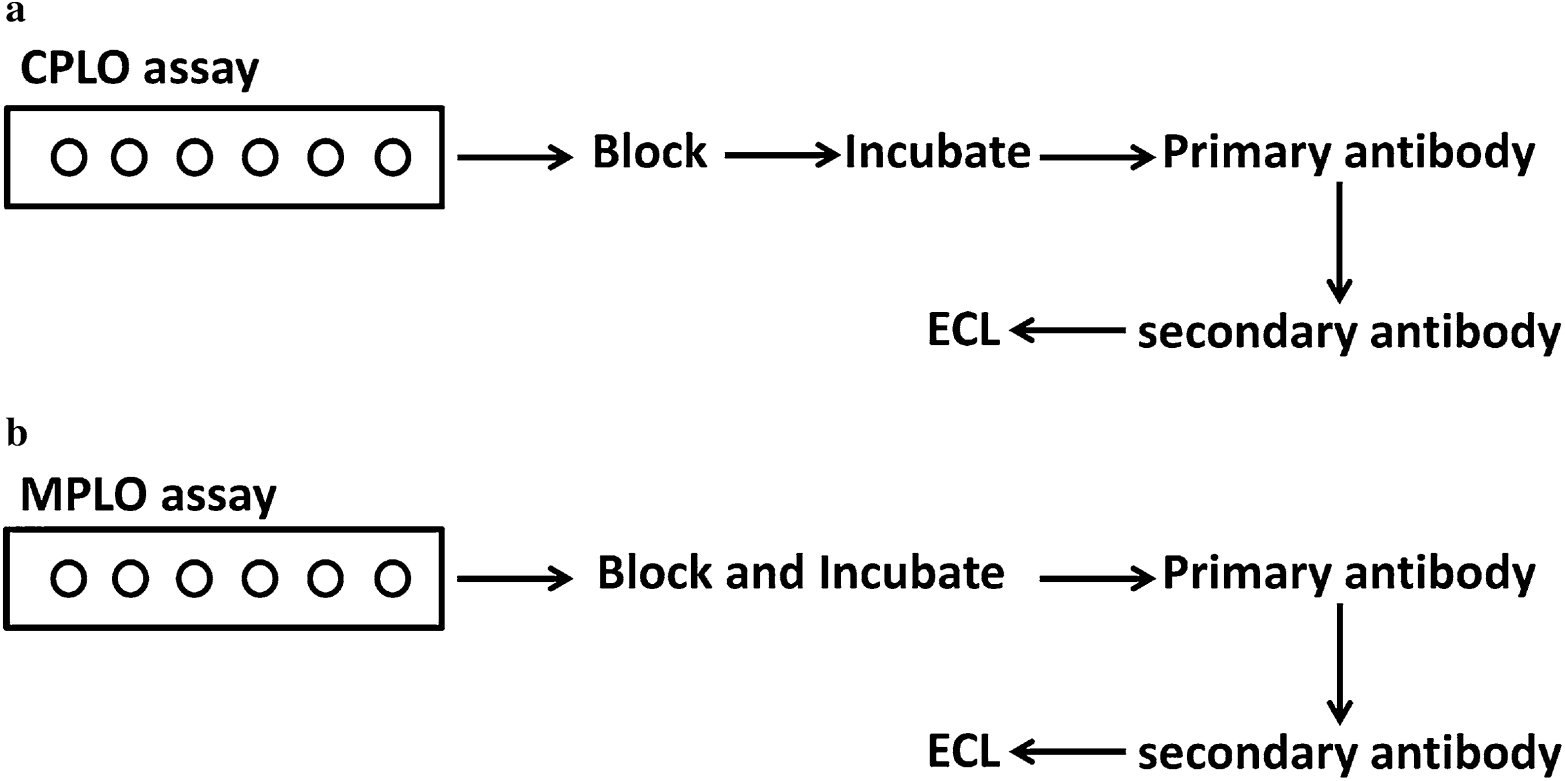
Schematic overview of the protein lipid overlay (PLO) assay. **a** Schematic overview of the classical protein lipid overlay (CPLO) assay. Different types or concentrations of lipids are spotted onto a PVDF membrane, blocked with PBST blocking buffer comprising 0.1% Tween 20 (v/v) and BSA (3%, m/v) or 0.1% Tween 20 (v/v) and skimmed milk (5%, m/v), then incubated with epitope-tagged (GST, MBP, His, etc.) proteins of interest at concentrations of 1 − 5 µg/mL. After washing and incubation with primary and secondary antibodies, proteins bound to the membrane are detected by enhanced chemiluminescence (ECL) according to the manufacturer’s instructions. **b** Schematic overview of the modified protein lipid overlay (MPLO) assay. Different types or concentrations of lipids are spotted onto a PVDF membrane, and the membrane is simultaneously blocked and incubated with epitope-tagged (GST, MBP, His, etc.) proteins of interest in PBST blocking buffer comprising 0.1% Tween 20 (v/v) and BSA (3%, m/v) or 0.1% Tween 20 (v/v) and skimmed milk (5%, m/v). After washing and incubation with primary and secondary antibodies, proteins bound to the membrane are detected by enhanced chemiluminescence (ECL) according to the manufacturer’s instructions. More details are provided in “Materials and methods”

In the CPLO assay, lipids were dissolved and spotted onto a PVDF membrane, dried, and blocked with blocking proteins (BSA or skimmed milk). The membrane was incubated with epitope-tagged lipid-binding proteins in blocking buffer, washed, incubated with primary antibody, then secondary antibody, and a signal was detected if epitope-tagged proteins interacted with lipids (Fig. 1a). In the assay, some lipids with a smaller head group displayed poor repeatability and low sensitivity (data not shown). We inferred that the blocking proteins in the blocking buffer may block some lipids nonspecifically, thereby limiting the lipid-binding proteins to contact membrane-bound lipids, lowering or eliminating the signal from lipid–protein binding. In MPLO assays, a blocking step was added to the incubation step (Fig. 1b), during which blocking proteins and lipid-binding proteins could bind lipids competitively in a dynamic process, thereby increasing the chances for lipid-binding proteins to contact lipids. Additionally, membranes were blocked with blocking proteins via non-specific binding. The MPLO assay displayed greatly improved repeatability and sensitivity compared with the CPLO assay, as demonstrated by the verification experiments described below.

#### PI3P and G302

**Fig. 2:**
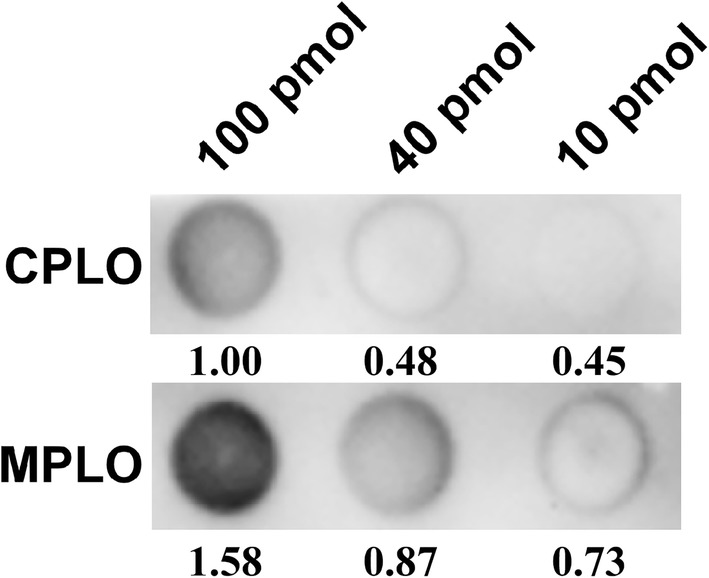
Comparison of CPLO and MPLO assays for the interaction between PI3P and G302. Assays were carried out between PI3P and its commercially available interacting protein G302 (Echelon). The amount of PI3P spotted onto a PVDF membrane is shown at the top. The upper and lower lanes show the interactions between PI3P and G302 using CPLO and MPLO assays, respectively. The amount of PI3P-binding G302 is shown on the bottom of the signal. *PI3P* phosphatidylinositol-3-phosphate. The strips of CPLO and MPLO assays were performed exposure simultaneously using the same ECL reagent and the same settings

To gain insight into differences between the MPLO and CPLO assays, we conducted binding assays between PI3P and its commercially available binding protein G302. CPLO and MPLO assays were carried out simultaneously in almost all procedures to exclude all factors that may influence the results. The only difference was that the membrane spotted with PI3P in the CPLO assay was first blocked using BSA blocking buffer for 1 h, then incubated with G302 in BSA blocking buffer for another hour, whereas in the MPLO assay the membrane spotted with PI3P was incubated with G302 directly in BSA blocking buffer for 1.5 h. During detection, both CPLO and MPLO membranes were exposed simultaneously to avoid variation in exposure to the ECL reagent. Our results showed that the MPLO assay provided a much stronger signal than the CPLO assay, as shown in Fig. 2, and Additional file 1: Figure S1A and B. The negative control assays between PI3P and OST1, and between solvent 1 and G302 were performed, which showed that PI3P did not bind OST1, and G302 did not bind solvent 1 (Additional file 1: Figure S2A and B). These results suggest that PI3P specifically binds G302, and this binding signal was enhanced in a MPLO assay compared with a CPLO assay.

### Free unsaturated fatty acids and the AHA2 C-terminus

**Fig. 3:**
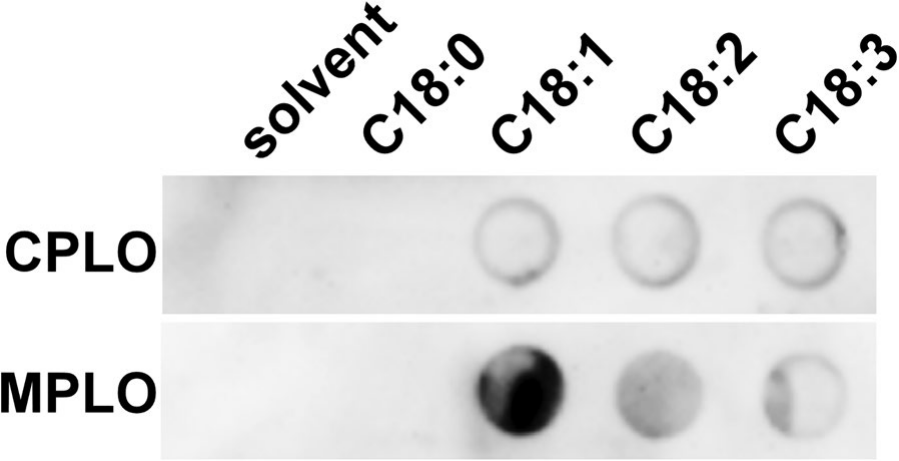
Comparison of CPLO and MPLO assays for the interaction between C18:1, C18:2, and C18:3 with AHA2 C-terminus. C18:0, C18:1, C18:2, and C18:3 were dissolved in 1:1 dichloromethane:MeOH and spotted onto a PVDF membrane. Solvent alone served as a negative control. The amount spotted was 5 nmol for each lipid, and lipids in each spot are shown at the top. AHA2 C-terminus was extracted and purified from *Escherichia coli* with His-epitope tag. The upper and lower lanes show detection using CPLO and MPLO assays, respectively. The strips of CPLO and MPLO assays were performed exposure simultaneously using the same ECL reagent and the same settings

The PM H^+^-ATPase is an important ion pump in plant cells for plant growth and development. By coupling ATP hydrolysis and proton transport, PM H^+^-ATPase establishes a membrane potential for secondary transporters to transporting many ions and nutrients [10]. In our previous study, we found that endogenous oleic acid (C18:1), linoleic acid (C18:2) and linolenic acid (C18:3) can stimulate PM H^+^-ATPase activity in Arabidopsis, and interaction assays confirmed that all three lipids bound to the C-terminus of the PM H^+^-ATPase AHA2 protein [13]. Fatty acids were dissolved in 1:1 dichloromethane:MeOH and the MPLO assay was performed. Other solvents, such as dichloromethane, chloroform, solvent 1, and solvent 2 are also applicable for dissolving fatty acids. We first examined the interaction between the three lipids and the PM H^+^-ATPase AHA2 C-terminus (amino acids 849–948) using CPLO assays, but the signal was weak and suffered from low repeatability (Fig. 3, Additional file 1: Figure S3A and B). After several repeated experiments to overcome this issue, we realized that the PLO assay procedure is very similar to that of western blot assays; however, there is obviously a large difference in size between membrane-bound lipids in PLO assays and membrane-bound proteins in western blot assays, and this may affect repeatability. We inferred that smaller lipids with a lower molecular mass and a smaller head group may be spatially blocked by proteins in blocking buffer in an unspecific manner, which may disrupt interactions between lipids and their binding proteins. To avoid this disturbance by the blocking proteins, we modified the procedure by combining blocking and incubation steps. Our results showed that the MPLO assay did indeed greatly improve repeatability for interactions between C18:1, C18:2, and C18:3 lipids with the AHA2 C-terminus, without increasing the background signal (Fig. 3, Additional file 1: Figure S3A and B). We also performed control assay between C18:1, C18:2, C18:3 and the AHA2 central loop, which showed no binding signal either in a CPLO assay or in a MPLO assay (Additional file 1: Figure S4A and B). These results indicate that the MPLO assay improved assay repeatability compared with the PLO assay.

### PG and AtROP6

**Fig. 4:**
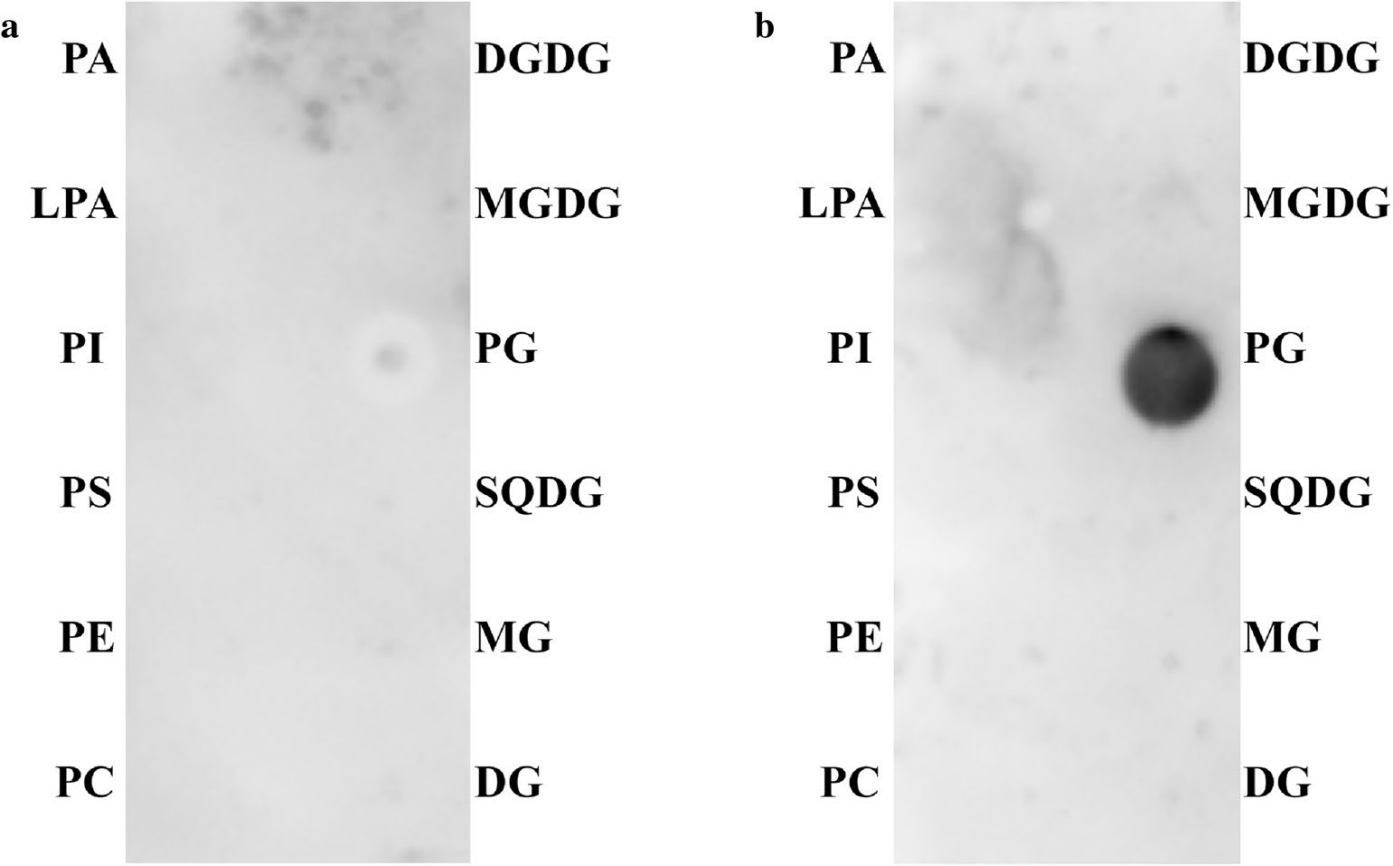
Comparison of CPLO and MPLO assays for the interaction between PG and recombinant protein AtROP6. **a** Interaction between PG and recombinant protein AtROP6 using the CPLO assay. **b** Interaction between PG and recombinant protein AtROP6 using the MPLO assay. DGDG, MGDG, PG, SQDG, and MG were dissolved in MeOH, DG was dissolved in chloroform, and the other lipids were dissolved in solvent 1 (65:35:8 chloroform:MeOH:H_2_O). The lipids were spotted a PVDF membrane and the lipid amount spotted was 5 nmol for each lipid. Lipids in each spot are shown on the left and right. AtROP6 was extracted and purified from *Escherichia coli* with His-epitope tag. *PA* phosphatidic acid, *LPA* lysophosphatidic acid, *PI* phosphatidylinositol, *PS* phosphatidylserine, *PE* phosphatidylethanolamine, *PC* phosphatidylcholine, *DGDG* digalactosyldiacylglycerol, *MGDG* monogalactosyldiacylglycerol, *PG* phosphatidylglycerol, *SQDG* sulfoquinovosyl diacylglycerol, *MG* monoglyceride, *DG* diglyceride. The strips of CPLO and MPLO assays were performed exposure simultaneously using the same ECL reagent and the same settings

Rho-related GTPases (ROPs), containing family members of AtROP1 to AtROP11 in Arabidopsis, play essential roles in many cell polarity-involved cellular activities, such as cell division and polarization [5]. AtROP6 is involved in modulating cell polarity and polar cell growth in vegetative cells in Arabidopsis (Craddock et al. 2012). In our previous study, we found that AtROP6 binds specifically to PG in PLO assay, liposome sedimentation assay, and MST assay [12]. In PLO assay between PG and AtROP6, the CPLO assay gave a weak signal with low repeatability, whereas the MPLO assay gave a stronger and more stable signal (Fig. 4a, b, Additional file 1: Figure S5–D). The negative control experiments between PG and CKL2, and between AtROP6 and lipid solvents showed that PG did not bind CKL2, and lipid solvents had no interaction with AtROP6 (Additional file 1: Figure S5E and F). These results suggest that the MPLO assay improves the signal from interaction between PG and its binding protein AtROP6 compared with the CPLO assay.

### PS and the AHA2 C-terminus

**Fig. 5:**
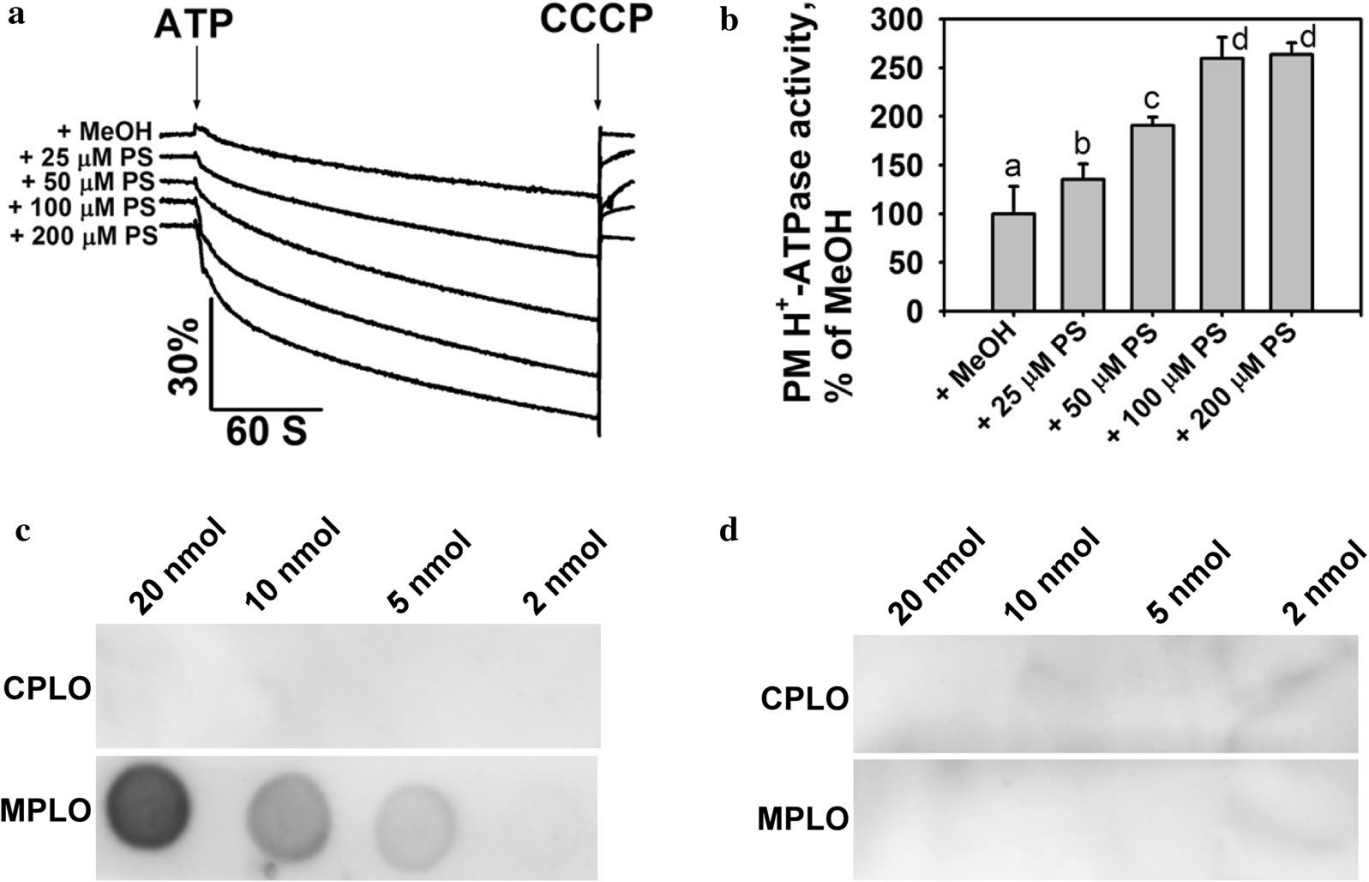
PLO assays of the interaction between PS and the AHA2 C-terminus. **a** Analysis of PM H^+^-ATPase activity in vesicles of Arabidopsis Col-0. The indicated amount of PS or MeOH solvent (control) was added, and PM H^+^-ATPase activity was initiated by addition of 3 mM ATP. Carbonyl cyanide m-chlorophenyl hydrazine (CCCP; 10 mM) was applied to collapse the pH gradient. **b** Comparison of PM H^+^-ATPase activities in **a**. **c** Interaction between PS and AHA2 C-terminus using CPLO and MPLO assays, respectively. PS was dissolved in 65:35:8 chloroform:MeOH:H_2_O and was spotted a PVDF membrane. The amount of PS spotted is shown at the top. The upper lane shows the interaction between PS and the AHA2 C-terminus using the CPLO assay. The lower lane shows the interaction between PS and the AHA2 C-terminus using the MPLO assay. The strips of CPLO and MPLO assays were performed exposure simultaneously using the same ECL reagent and the same settings. **d** Interaction between PS and AHA2 central loop using CPLO and MPLO assays, respectively. PS was dissolved in solvent 1 and spotted onto PVDF membrane. The amount of PS spotted on the membrane is shown at the top. The upper lane shows the interaction between PS and the AHA2 central loop using the CPLO assay. The lower lane shows the interaction between PS and the AHA2 central loop using the MPLO assay. The strips of CPLO and MPLO assays were performed exposure simultaneously using the same ECL reagent and the same settings. Data in **b** are mean ± standard deviation (SD) from five replicates. Student’s t-tests were used to analyze the statistical significance; significant differences (p ≤ 0.05) in **b** are indicated by different lower-case letters. The experiments in **a** and **b** were performed independently at least three times

PS activated PM H^+^-ATPase activity in previous studies [17]; however, whether PS directly binds to PM H^+^-ATPase and affects its activity remains unknown. Herein, we performed PM H^+^-ATPase activity assays to investigate the effect of PS on PM H^+^-ATPase activity. PS or 0.1% MeOH (v/v) was pre-incubated with plasma membrane vesicles and PM H^+^-ATPase activity was measured. As shown in Fig. 5a, b, exogenous addition of PS to vesicles caused a significant increase in H^+^-transport activity compared with the MeOH control. To understand how PS regulates PM H^+^-ATPase activity and identify which part of PM H^+^-ATPase AHA2 is involved in this regulation, we performed PLO binding assays between PS and AHA2 peptides (the AHA2 C-terminus consisting of amino acids 849–948, and the AHA2 central loop consisting of amino acids 321–620). MPLO and CPLO assays were performed simultaneously, and signals were detected. As shown in Fig. 5c, d and Additional file 1: Fig. 6A–D, the MPLO assay showed that PS physically interacted with the C-terminus of AHA2, but not its central loop. However, the CPLO assay indicated that PS did not bind either the AHA2 C-terminus or its central loop. This result suggests that membrane-bound PS may be blocked by blocking proteins in skimmed milk in the CPLO assay, whereas the AHA2 C-terminus and proteins in skimmed milk compete for binding to PS in a dynamic process in the MPLO assay.

## Discussion

PVDF membranes are widely used in western blot assays to transfer proteins from SDS-PAGE gels for protein detection and characterization [22]. PVDF has some outstanding properties, such as thermal stability, a smooth surface, high mechanical strength, low protein adsorption, and resistance to ultraviolet radiation [38]. However, hydrophobicity is the key factor determining its application in western blot analysis for binding between proteins [25]. Lipids are small amphiphilic molecules with a polar head group and nonpolar acyl chains [9]. We inferred that PVDF membranes would mainly bind to the nonpolar acyl chains of lipids, while the polar head groups would likely extend away from the membrane, and may be involved in recognition and binding to proteins. In both PLO assays and western blot assays, the purpose of blocking with blocking buffer is to block non-specific areas of the membrane using proteins in skimmed milk, BSA, or other reagents. Two situations may occur in PLO assays; the size and conformation of membrane-bound lipids may be favorable for extending away from the membrane, so that blocking lipids with blocking proteins is inefficient, and they only bind the membrane and reduce the background signal; alternatively, the size and conformation of membrane-bound lipids may be unfavorable for extending away from the membrane, so that blocking proteins may efficiently block and hinder membrane-bound lipids nonspecifically, effectively preventing lipid-binding proteins from contacting lipids, resulting in a poor signal, or no signal at all. Compared with membrane-bound proteins in western blot assays, membrane-bound lipids may not be able to extend far away from the PVDF membrane, so that skimmed milk or BSA proteins in blocking buffer can disrupt interactions between lipids and their binding proteins if a separate blocking step is carried out before the incubation step.

To ensure the specificity of this method to exclude the nonspecific binding of lipids and proteins caused by removing the blocking step, we constructed protein of MPK6, which has been reported to bind PA, not PC, PE, PG, PI, PS and DG [37]. We performed a MPLO assay between MPK6 and PA, PC, PE, PG, PI, PS and DG, which showed that MPK6 specifically interacted PA, not PC, PE, PG, PI, PS and DG (Additional file 1: Figure S7). These results suggest that a MPLO assay, compared with a CPLO assay, would not cause the nonspecific bindings of lipids and proteins. We still could not rule out the nonspecific bindings of lipids and proteins both in a MPLO assay and a CPLO assay, which need further investigation to improve this technology. To rule out the nonspecific bindings in a PLO assay, some other techniques, such as MST, NMR and liposome sedimentation assay were needed to further verify the interactions between lipids and proteins.

In addition to the amount of lipid, different exposure time settings usually show different signal intensities. In this study, signals from membranes of CPLO and MPLO were captured simultaneously using a digital imaging system (instrument: viber lourmat, fusion solo), which could also be performed by exposing a PVDF membrane to a photographic film in a traditional detection. Although automatic exposure can be performed during a digital imaging, one needs to adjust the increase or decrease of the exposure time if the signal is too weak or too strong. To exclude the influence of exposure time on signal intensity when comparing a CPLO assay and a MPLO assay, the membranes of CPLO and MPLO were exposed simultaneously, so they have the same exposure time. The exposure time may be affected by the binding affinity, ECL reagent, and background signal noise. Therefore, in a digital imaging or a traditional photographic film, repeated experiments are required to rule out the possibility that a longer exposure time may cause membrane to be overexposed and a shorter exposure time may cause the signal to be undetectable.

To explore this further, we tried to calculate the theoretical distances between atoms in the glycerol skeleton and atoms in the polar head group of the lipid. The molecular structures of PI3P, PS, and PG were drawn (Additional file 1: Figures S8A–C) and transferred to the ChemBio3D module for structure optimization. After optimization by the MM2 force field in ChemBio3D Ultra 12.0 (Additional file 1: Figures S9A–C), distances between atoms were measured (Additional file 1: Tables S1–S3). From our calculations, the maximum distances for PI3P, PS, and PG were < 15 Å. However, for a protein with α-helical secondary structure, there are 3.6 amino acids per helical turn, and the pitch of the α-helix is ~ 0.54 nm (5.4 Å). Thus, a protein with dozens or hundreds of amino acids may not be easily blocked by blocking proteins, whereas smaller lipids may be blocked more easily. These results may indicate differences between membrane-bound lipids in PLO assays and membrane-bound proteins in western blot assays, which supports the idea that blocking proteins may interfere with lipid–protein interactions if a separate blocking step is carried out before the incubation step. Therefore, MPLO assays with a combined blocking and incubation step may avoid interference by blocking proteins, yielding a better signal without enhancing background noise.

In PLO assays, either increasing lipid or protein concentration, or increasing incubation time may cause changes in lipid–protein binding signal detected, which needs further investigation. In this study, the interaction between lipid and its binding protein has a dose-dependent effect and increasing lipid concentration would have a stronger signal detected; however, the binding signal would not change after reaching saturation or would show overexposed pattern depending on a specific lipid–protein binding. Increasing protein concentration is not suggested in this study, because false signal might be caused and background signal would be increased. We did not see a significant influence of increasing incubation time on a binding signal detected in this study, which need further investigation. Although the effect of lipid concentration, protein concentration and incubation time on binding signals detected has no obvious difference between CPLO assays and MPLO assays in this study, more detailed research is needed in the further study. Moreover, in a MPLO assay, the concentration of lipid binding protein (1–5 µg/mL) is much greater than the concentration of blocking proteins (30 mg/mL BSA or 50 mg/mL skimmed milk), which indicate that the combination of a blocking step and an incubation step in a MPLO assay would have little chance to cause more false positive results compared with a CPLO assay. We could not rule out the false positive results both in a MPLO assay and in a CPLO assay due to the technical limitation of PLO assay, which need further investigation to improve it. Some other techniques, such as MST, NMR, and liposome sedimentation assay, are suggested to verify the interactions between lipids and their binding proteins.

Overall, the MPLO assay greatly improves the signal compared with CPLO assays. The MPLO assay should be applicable to a variety of lipids, and may help to reveal their functions in plants and other organisms.

## Conclusions

The MPLO assay developed herein is more sensitive and efficient for studying lipid–protein interactions than CPLO assays. In addition to greatly improving the signal from interactions between lipids and their interacting proteins, our MPLO assay may be suitable for investigating lipid–protein interactions that cannot be detected using traditional CPLO assays. Therefore, our MPLO method will likely expand the study of lipids and their binding proteins, and reveal new functions for lipids in different organisms.

## Supplementary information

**Additional file 1: Figure S1.** The replicate experiments for the interaction assay between PI3P and G302 using CPLO and MPLO assays, respectively. **Figure S2.** The interaction assays between PI3P and OST1, and between G302 and solvent 1 using CPLO and MPLO assays, respectively. **Figure S3.** The replicate experiments for the interaction assay between C18:1, C18:2, C18:3 and AHA2 C-terminus using CPLO and MPLO assays, respectively.**Figure S4.** The interaction assay between C18:1, C18:2, C18:3 and AHA2 central loop using CPLO and MPLO assays, respectively. **Figure S5.** The replicate experiments for the interaction assays between PG and AtROP6 using CPLO and MPLO assays, respectively, and the interaction assays between PG and CKL2, and between AtROP6 and lipid solvents using CPLO and MPLO assays, respectively. **Figure S6.** The replicate experiments for the interaction assay between PS and AHA2 peptides (C-terminus and central loop) using CPLO and MPLO assays, respectively. **Figure S7.** The interaction assay between PA and MPK6 using MPLO assay. **Figure S8.**. Structures of PI3P, PS, and PG drawn using ChemBioDraw. Figure S9. Structures of PI3P, PS, and PG transferred to ChemBio3D after MM2 optimization. **Table S1.** Distances between atoms in the glycerol skeleton and the lipid polar head group measured by ChemBio3D for PI3P. **Table S2.** Distances between atoms in the glycerol skeleton and the lipid polar head group measured by ChemBio3D for PS. **Table S3.** Distances between atoms in the glycerol skeleton and the lipid polar head group measured by ChemBio3D for PG. **Table S4.** Primers used for plasmid construction.

## Data Availability

The datasets supporting the conclusions of this article are included within the article and its additional files.

## Abbreviations

PLO: Protein lipid overlay
CPLO: Classical protein lipid overlay
MPLO: Modified protein lipid overlay
PM H^+^-ATPase: Plasma membrane H^+^-ATPase
NMR: Nuclear magnetic resonance
MST: Microscale thermophoresis
ITC: Isothermal titration calorimetry
SPR: Surface plasmon resonance
PC: Phosphatidylcholine
PE: Phosphatidylethanolamine
PG: Phosphatidylglycerol
PA: Phosphatidic acid
DG: Diglyceride
PI(3)P: Phosphatidylinositol 3-phosphate
LPA: Lysophosphatidic acid
MG: Monoglyceride
MGDG: Monogalactosyldiacylglycerol
DGDG: Digalactosyldiacylglycerol
SQDG: Sulfoquinovosyl diacylglycerol
PS: Phosphatidylserine
PI: Phosphatidylinositol
CCCP: Carbonyl cyanide 3-chlorophenylhydrazone
MS: Murashige and Skoog salts
ECL: Enhanced chemiluminescence
