# Supplementary material for: An improved protein lipid overlay assay for studying lipid–protein interactions
Source: Plant Methods. 2020 Mar 6;16:33. doi: 10.1186/s13007-020-00578-5 (PMC7060618; doi:10.1186/s13007-020-00578-5)
Supplement: Supplementary file 1 — Additional file 1: Figure S1. The replicate experiments for the interaction assay between PI3P and G302 using CPLO and MPLO assays, respectively. Figure S2. The interaction assays between PI3P and OST1, and between G302 and solvent 1 using CPLO and MPLO assays, respectively. Figure S3. The replicate experiments for the interaction assay between C18:1, C18:2, C18:3 and AHA2 C-terminus using CPLO and MPLO assays, respectively.Figure S4. The interaction assay between C18:1, C18:2, C18:3 and AHA2 central loop using CPLO and MPLO assays, respectively. Figure S5. The replicate experiments for the interaction assays between PG and AtROP6 using CPLO and MPLO assays, respectively, and the interaction assays between PG and CKL2, and between AtROP6 and lipid solvents using CPLO and MPLO assays, respectively. Figure S6. The replicate experiments for the interaction assay between PS and AHA2 peptides (C-terminus and central loop) using CPLO and MPLO assays, respectively. Figure S7. The interaction assay between PA and MPK6 using MPLO assay. Figure S8.. Structures of PI3P, PS, and PG drawn using ChemBioDraw. Figure S9. Structures of PI3P, PS, and PG transferred to ChemBio3D after MM2 optimization. Table S1. Distances between atoms in the glycerol skeleton and the lipid polar head group measured by ChemBio3D for PI3P. Table S2. Distances between atoms in the glycerol skeleton and the lipid polar head group measured by ChemBio3D for PS. Table S3. Distances between atoms in the glycerol skeleton and the lipid polar head group measured by ChemBio3D for PG. Table S4. Primers used for plasmid construction. [file 13007_2020_578_MOESM1_ESM.docx]

Additional file 1: Figure S1. The replicate experiments for the interaction assay between PI3P and G302 using CPLO and MPLO assays, respectively.


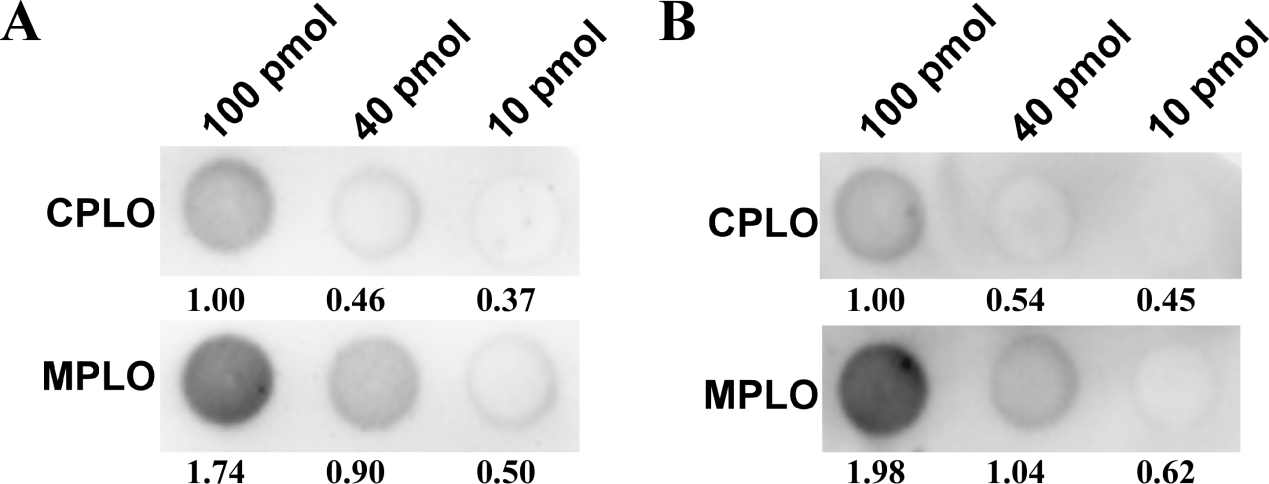


(A) The interaction assay between PI3P and G302 using CPLO and MPLO assays, respectively.

(B) The interaction assay between PI3P and G302 using CPLO and MPLO assays, respectively.

PI3P was dissolved in solvent 1 and spotted onto a PVDF membrane. Each spot of PI3P amount is shown at the top. The amount of PI3P-binding G302 is shown on the bottom of the signal. The strips of CPLO and MPLO assays in each replicate experiment were performed exposure simultaneously using the same ECL reagent and the same settings.

Additional file 1: Figure S2. The interaction assays between PI3P and OST1, and between G302 and solvent 1 using CPLO and MPLO assays, respectively.


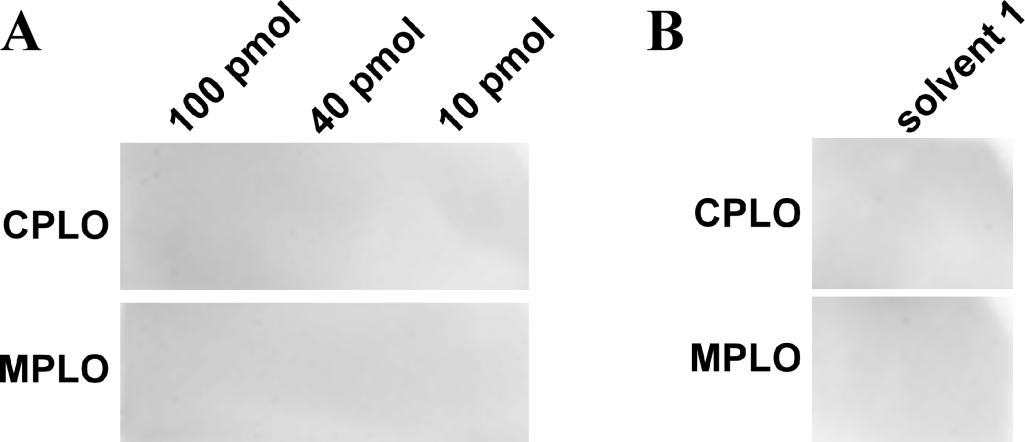


(A) The interaction assay between PI3P and OST1 using CPLO and MPLO assays, respectively.

(B) The interaction assay between G302 and solvent 1 using CPLO and MPLO assays, respectively.

PI3P was dissolved in solvent 1 and spotted onto a PVDF membrane. Each spot of PI3P amount is shown at the top. The upper and lower lanes show detection using CPLO and MPLO assays, respectively. The strips of CPLO and MPLO assays in each replicate experiment were performed exposure simultaneously using the same ECL reagent and the same settings. Solvent 1: chloroform:methanol: H_2_O 65:35:8 (v/v/v)

Additional file 1: Figure S3. The replicate experiments for the interaction assay between C18:1, C18:2, C18:3 and AHA2 C-terminus using CPLO and MPLO assays, respectively.


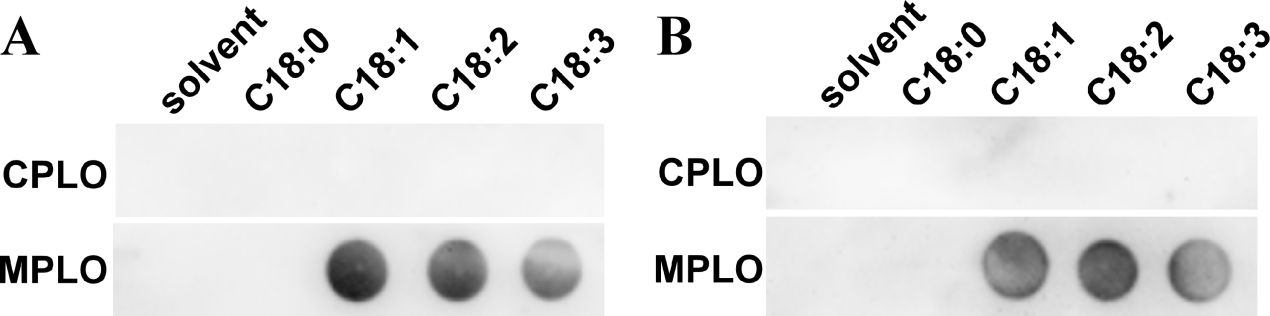


(A) The interaction assay between C18:1, C18:2, C18:3 and AHA2 C-terminus using CPLO and MPLO assays, respectively.

(B) The interaction assay between C18:1, C18:2, C18:3 and AHA2 C-terminus using CPLO and MPLO assays, respectively.

C18:0, C18:1, C18:2, and C18:3 were dissolved in 1:1 dichloromethane:methanol and spotted onto a PVDF membrane. Solvent alone served as a negative control. The amount spotted was 5 nmol for each lipid, and lipid in each spot was shown at the top. AHA2 C-terminus was extracted and puriﬁed from *Escherichia coli* with His-epitope tag. The strips of CPLO and MPLO assays were performed exposure simultaneously using the same ECL reagent and the same settings.

Additional file 1: Figure S4. The interaction assay between C18:1, C18:2, C18:3 and AHA2 central loop using CPLO and MPLO assays, respectively.


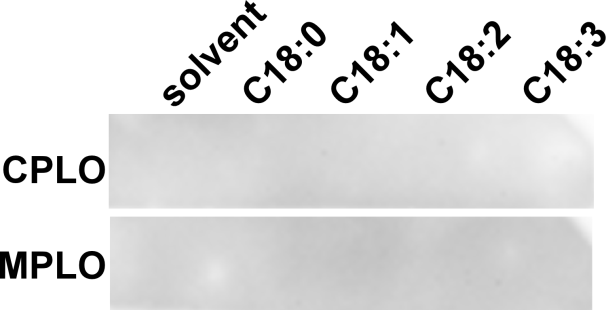


C18:0, C18:1, C18:2, and C18:3 were dissolved in 1:1 dichloromethane:methanol and spotted onto a PVDF membrane. Solvent alone served as a negative control. The amount spotted was 5 nmol for each lipid, and lipid in each spot was shown at the top. AHA2 central loop was extracted and puriﬁed from *Escherichia coli* with His-epitope tag. The upper and lower lanes show detection using CPLO and MPLO assays, respectively. The strips of CPLO and MPLO assays were performed exposure simultaneously using the same ECL reagent and the same settings.

Additional file 1: Figure S5. The replicate experiments for the interaction assays between PG and AtROP6 using CPLO and MPLO assays, respectively, and the interaction assays between PG and CKL2, and between AtROP6 and lipid solvents using CPLO and MPLO assays, respectively.


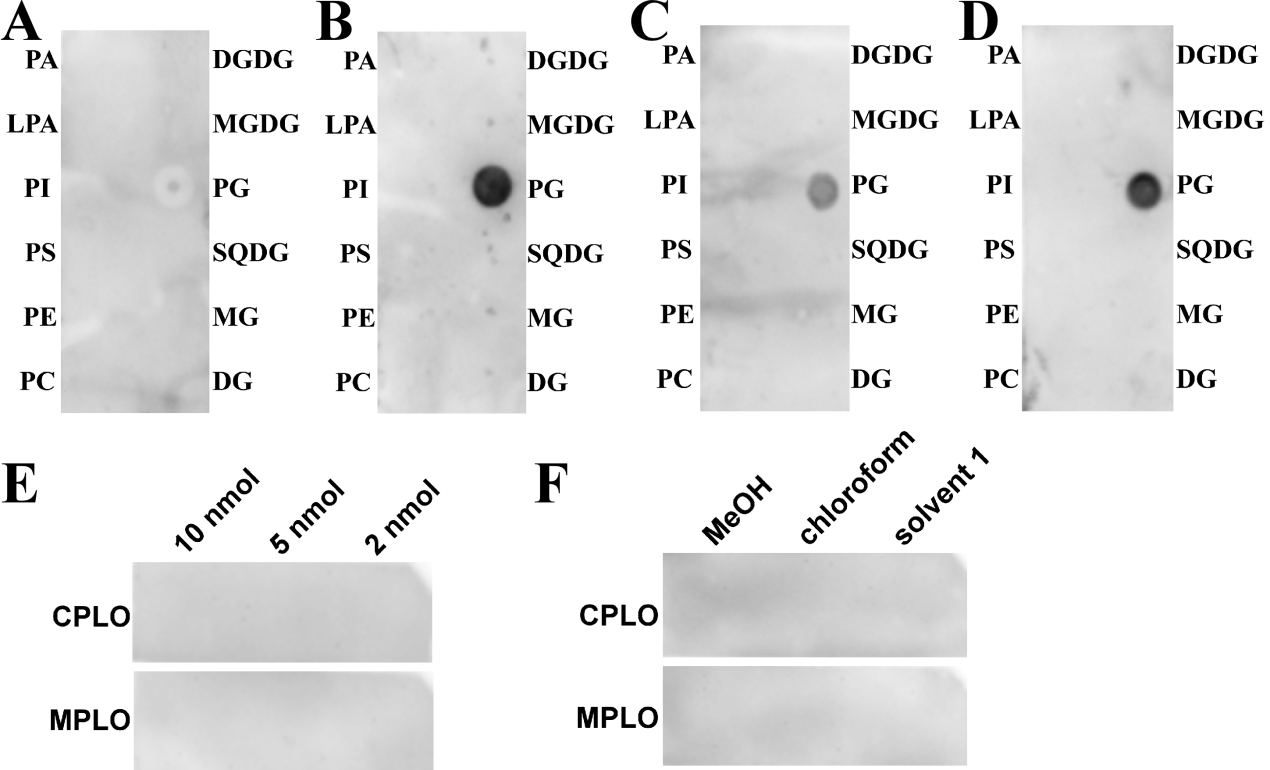


(A) The interaction assay between PG and AtROP6 using CPLO assays.

(B) The interaction assay between PG and AtROP6 using MPLO assays.

(C) The interaction assay between PG and AtROP6 using CPLO assays.

(D) The interaction assay between PG and AtROP6 using MPLO assays.

(E) The interaction assay between PG and CKL2 using CPLO and MPLO assays, respectively.

(F) The interaction assay between various solvents and AtROP6 using CPLO and MPLO assays, respectively.

DGDG, MGDG, PG, SQDG, and MG were dissolved in MeOH, DG was dissolved in chloroform, and the other lipids were dissolved in solvent 1. The lipids were spotted onto a membrane, and the amount of lipid spotted in (A), (B), (C) and (D) was 5 nmol, and the amount of lipid spotted in (E) was shown at the top. AtROP6 and CKL2 were extracted and puriﬁed from *Escherichia coli* with His-epitope tags. The strips of CPLO and MPLO assays were performed exposure simultaneously using the same ECL reagent and the same settings.

Additional file 1: Figure S6. The replicate experiments for the interaction assay between PS and AHA2 peptides (C-terminus and central loop) using CPLO and MPLO assays, respectively.


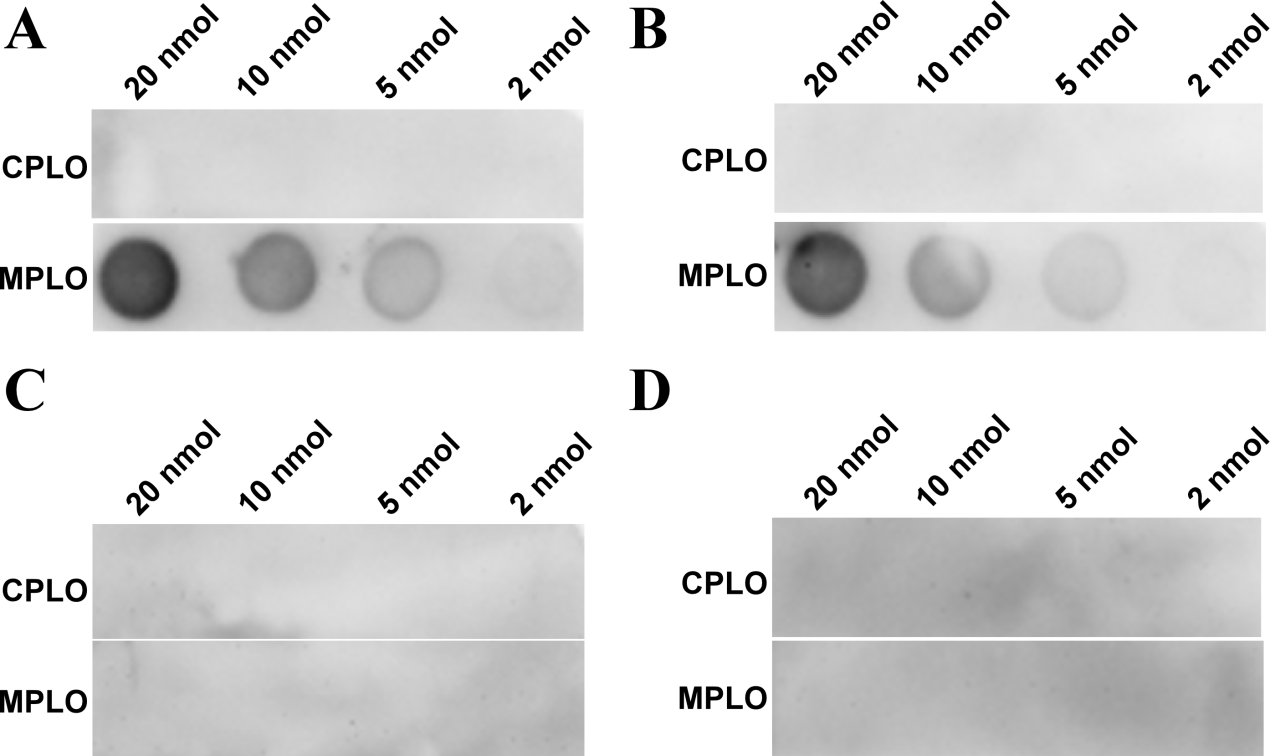


(A) The interaction assay between PS and AHA2 C-terminus using CPLO and MPLO assays, respectively.

(B) The interaction assay between PS and AHA2 C-terminus using CPLO and MPLO assays, respectively.

(C) The interaction assay between PS and AHA2 central loop using CPLO and MPLO assays, respectively.

(D) The interaction assay between PS and AHA2 central loop using CPLO and MPLO assays, respectively.

PS was dissolved in solvent 1 and spotted onto PVDF membrane. The amount of PS spotted on the membrane was shown at the top. AHA2 C-terminus and AHA2 central loop were extracted and puriﬁed from *Escherichia coli* with His-epitope tags. The strips of CPLO and MPLO assays in each replicate experiment were performed exposure simultaneously using the same ECL reagent and the same settings.

Additional file 1: Figure S7. The interaction assay between PA and MPK6 using MPLO assay.


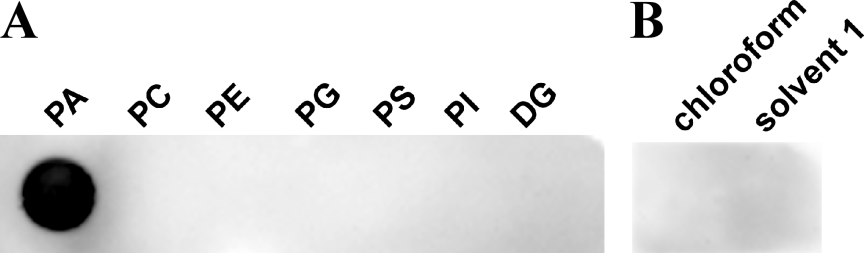


(A) The interaction assay between various lipids and MPK6 using MPLO assay.

(B) The interaction assay between solvents and MPK6 using MPLO assay.

DG was dissolved in chloroform, and the other lipids were dissolved in solvent 1. The lipids were spotted onto a PVDF membrane, and the amount of each lipid spot is 2 nmol. MPK6 was extracted and puriﬁed from *Escherichia coli* with His-epitope tag.

Additional file 1: Figure S8. The structures of PI3P, PS and PG drawn in ChemBioDraw.


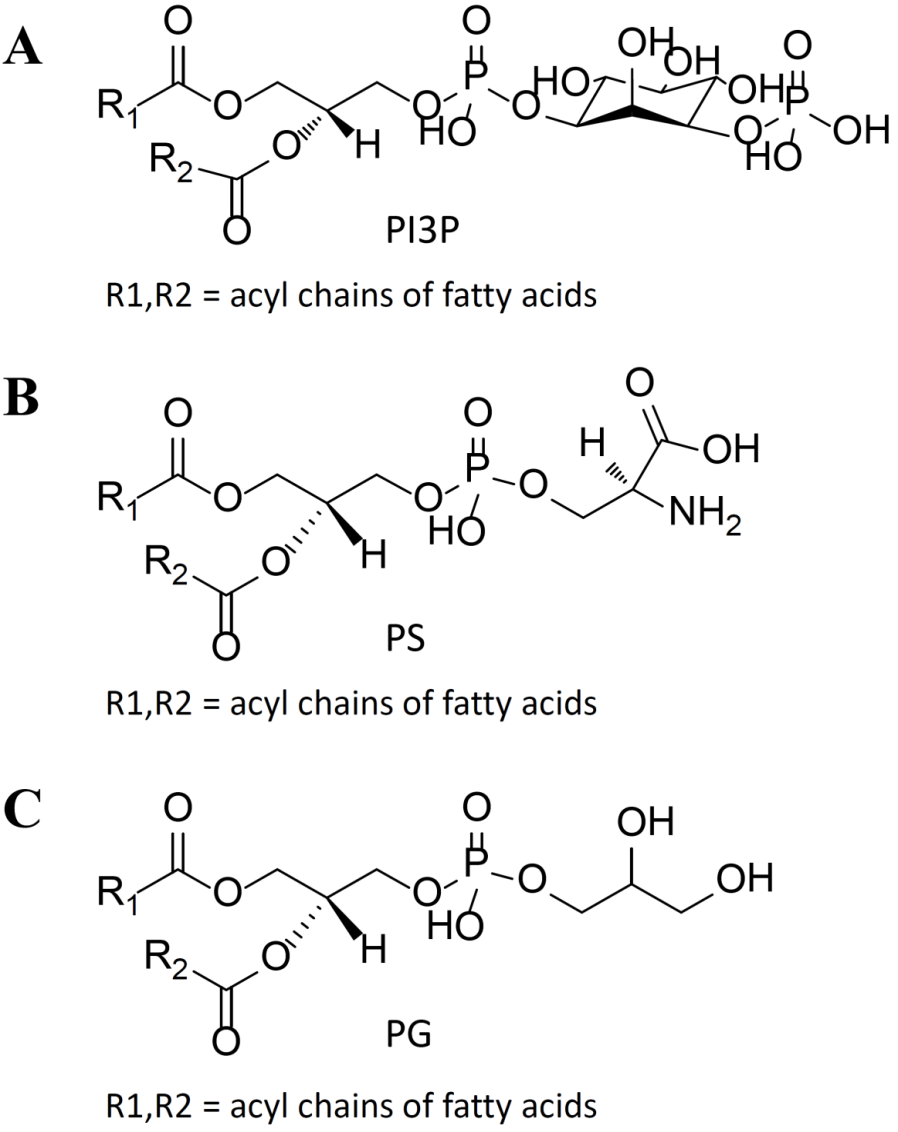


(A) The structure of PI3P drawn in ChemBioDraw.

(B) The structure of PS drawn in ChemBioDraw.

(C) The structure of PG drawn in ChemBioDraw.

Additional file 1: Figure S9. The structures of PI3P, PS and PG transferred to ChemBio3D after MM2 optimization.


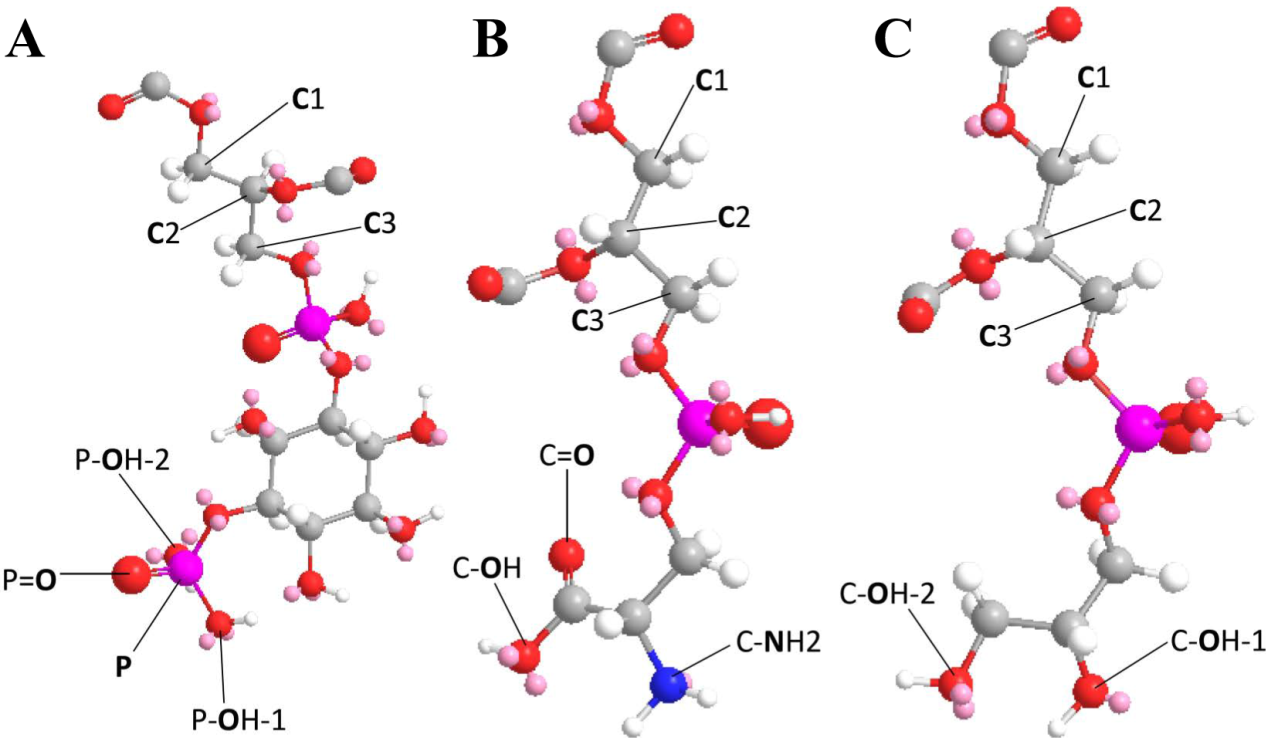


(A) The structures of PI3P transferred to ChemBio3D after MM2 optimization.

(B) The structures of PS transferred to ChemBio3D after MM2 optimization.

(C) The structures of PG transferred to ChemBio3D after MM2 optimization.

Additional file 1: Table S1. The distances between the atoms of the glycerol skeleton and the atoms of polar head group measured in ChemBio3D for PI3P.

| PI3P | |
| --- | --- |
| Two atoms measured | Distance (Å) |
| P - C1 | 9.9 |
| P=O - C1 | 10.2 |
| P-OH-1 - C1 | 11.3 |
| P-OH-2 - C1 | 9.9 |
| P - C2 | 9.5 |
| P=O - C2 | 9.9 |
| P-OH-1 - C2 | 10.7 |
| P-OH-2 - C2 | 9.5 |
| P - C3 | 8.1 |
| P=O - C3 | 8.6 |
| P-OH-1 - C3 | 9.3 |
| P-OH-2 - C3 | 8 |

Additional file 1: Table S2. The distances between the atoms of the glycerol skeleton and the atoms of polar head group measured in ChemBio3D for PS.

| PG | |
| --- | --- |
| Two atoms measured | Distance (Å) |
| C-OH-1 - C1 | 9.7 |
| C-OH-2 - C1 | 9.8 |
| C-OH-1 – C2 | 8.4 |
| C-OH-2 – C2 | 8.3 |
| C-OH-1 – C3 | 7.3 |
| C-OH-2 – C3 | 7.7 |

Additional file 1: Table S3. The distances between the atoms of the glycerol skeleton and the atoms of polar head group measured in ChemBio3D for PG.

| PS | |
| --- | --- |
| Two atoms measured | Distance (Å) |
| C-OH - C1 | 9.5 |
| C=O - C1 | 7.5 |
| C-NH2 – C1 | 9.7 |
| C-OH – C2 | 8.0 |
| C=O – C2 | 6.2 |
| C-NH2 – C2 | 8.4 |
| C-OH – C3 | 7.4 |
| C=O – C3 | 5.6 |
| C-NH2 – C3 | 7.3 |

Additional file 1: Table S4. Primers used for plasmids construction.

| Primer name | Sequence |
| --- | --- |
| L-Bf | CGGGATCCGCAGGAATGGATGTCCTGTGC |
| L**-**Er | CGGAATTCTTAGAGCACGATATCTGAAGCACC |
| C-Bf | CGGGATCCGCGTGGCTCAACTTGTTTGAGAAC |
| C-Er | CGGAATTCCTACACAGTGTAGTGACTG |
| R6-Bf | CGGGATCCATGAGTGCTTCAAGGTTT |
| R6-Hr | CCAAGCTTTCAGAGTATAGAACAACC |
| CKL2-Bf | CGGGATCCATGGAGCCGC GAGTCGGAAA |
| CKL2-Er | CGGAATTCCTAAAAATGCAGACTCTCGA |
| MPK6-Bf | CGGGATCCATGGACGGTGGTTCAGGTCA |
| MPK6-Hr | CCAAGCTTCTATTGCTGATATTCTGGAT |
